# Continuum Compressive Damage Modelling in Composites Using Genetic Algorithms and Nonlocal Averaging

**DOI:** 10.3390/polym17070902

**Published:** 2025-03-27

**Authors:** Johannes Reiner, Yun-Fei Fu

**Affiliations:** 1 School of Engineering, Faculty of Science Engineering and Built Environment, Deakin University, Geelong, VIC 3216, Australia; 2 Department of Mechanical Engineering, University of Alberta, Edmonton, AB T6G 1H9, Canada; yfu15@ualberta.ca

**Keywords:** finite element analysis, continuum damage mechanics, genetic algorithm, progressive damage

## Abstract

Continuum modelling of progressive damage in finite element analyses of fibre-reinforced polymers (FRPs) has become a popular tool because of its computational efficiency and ease of implementation. However, two of the major limitations are (i) mesh size and mesh orientation dependencies and (ii) the transparent determination of suitable input parameters. This study presents a combination of genetic algorithms (GA) with nonlocal continuum damage models to overcome these limitations. The use of GA provides an objective calibration process of input parameters, while nonlocal averaging of computed strain fields enables consistent damage evolution in FRPs irrespective of the underlying finite element mesh. The simulation of compact compression and open-hole compression tests on IM7/8552 carbon-fibre-reinforced polymers validates the calibration process and demonstrates the advantages of nonlocal damage modelling over conventional local approaches.

## 1. Introduction

The finite element (FE) simulation of progressive damage in fibre-reinforced polymer (FRP) composites is a challenging task owing to a range of interacting failure modes at various length scales. Over the past decades, many computational fracture/damage modelling techniques have been proposed. Generally, these techniques can be classified as discrete or continuum methods [1].

Discrete methods can realistically represent spatial discontinuities (cracks). The Cohesive Zone Method (CZM) [2] is a popular tool for inserting discrete cracks into FE simulations. Typically, cohesive contacts/elements are inserted along a predefined crack path, which is identified through experimental observations [3]. This means that the direction of crack growth must be known before the simulation, hence limiting the capabilities of simulating arbitrary crack growth. Alternatively, enrichment methods can be applied to account for discrete crack growth by means of partition of unity. Examples are the Extended Finite Element Method (X-FEM) [4], the Phantom Node Method (PNM) [5,6] and the Floating Node Method (FNM) [7]. All of the discrete methods have a high potential for realistic crack growth simulations; however, their complex implementation and high computational cost prevent their wider use.

The continuum representation of cracks in the form of Continuum Damage Mechanics (CDM) has proven to be an efficient and relatively simple FE modelling approach to account for stiffness degradation due to damage growth while maintaining the continuity of the FE mesh. Voids and cracks are smeared out by applying effective material properties [8]. Recent examples of the successful application of CDM to FRP composites are the simulation of centre-notched tension tests [9], impact [10] and compression-after-impact [11] tests, bi-axial tensile tests [12] and the virtual certification of FRP composites [13,14,15]. Note that, compared to investigations of FRP composites loaded in tension, CDM simulations of compressive load cases are rare due to the presence of challenging failure modes and a high sensitivity to fibre misalignment, which result in large variabilities [16].

The majority of developed CDM models compute stresses and strains locally without considering the state of neighbouring integration points. One significant limitation of these local CDM models is their mesh size and mesh orientation sensitivity [17,18]. If the direction of damage growth is known a priori, FE meshes can be aligned with this direction to enable realistic damage evolution [19]. Of course, such mesh alignment is a major intervention, which prevents the FE simulation of FRP composites where the damage path is not known, similar to the limitation on the CZM. Nonlocal CDM methods do not suffer from these limitations as stresses and strains are evaluated by taking into account integration points in a finite neighbourhood. This can be achieved by nonlocal averaging [20,21] or through gradient-based formulations [22]. As an example of a gradient-based method, the phase field technique [23,24] has gained popularity for simulating mesh-independent damage evolution in FRP composites.

Regardless of the underlying damage modelling technique, it is important to provide an objective and transparent strategy to determine FE input parameters [25] for progressive damage simulations. While some input data can be gathered from standard experimental tests, for example, elastic moduli and strength data, other damage input parameters such as fracture energies are less intuitive to determine. Furthermore, the transition from local to nonlocal simulations requires the adaptation of some input parameters. To avoid cumbersome trial-and-error calibration processes to determine these parameters, an objective and automated methodology is required.

If the computational modelling is efficient enough to generate large simulation datasets, data-driven calibration methods are a powerful strategy to find suitable input parameters. One example is the application of machine learning algorithms to calibrate CDM material models [16,26,27]. Genetic algorithms (GA) are an alternative method to identify FE inputs where fewer FE simulations are required compared to similar machine learning approaches [28].

This paper compares an efficient CDM model in local and nonlocal form to simulate progressive damage in FRP composites subjected to compressive loadings. First, FE input parameters for the local and nonlocal models are generated by GA in Section 2. The optimised input parameters are then validated against open-hole compression tests in Section 3 before a detailed discussion on mesh size and mesh orientation dependencies in Section 4.

The methodology presented here for GA-based parameter identification of local and nonlocal CDM material models paves the way towards tweak-free, objective calibration processes for simulating progressive damage in FRP composites. The results confirm the advantages of nonlocal CDM for achieving mesh-independent simulation results. The objective and automated calibration scheme removes the barriers about uncertain FE input parameters, in particular, for nonlocal CDM material models where not all input parameters can be directly linked to physical properties.

## 2. Genetic Algorithm for Parameter Calibration

This section will present an FE framework that is efficient enough for coupling with a data-driven calibration scheme by means of GA. It consists of the CDM material model in Section 2.1 and the FE models in Section 2.2 and can simulate progressive damage in FRP composites subjected to compressive loadings. Thereby, the CDM material model will be applied in local and nonlocal form to compare the two modelling approaches.

### 2.1. Continuum Damage Model CODAM2

The Composite Damage Model (CODAM2) is available as MAT219 in the commercial FE software LS-DYNA (as part of Ansys 2021 R2). The CODAM2 is a progressive damage model capturing the essence of damage evolution in composite plies [29] whereby nonlocal CDM can be easily activated by assigning an averaging radius within the material card, as explained in the following.

The strain-based material model evaluates damage as a function of equivalent strains. The longitudinal (fibre) equivalent strain $\varepsilon_{1}^{\mathrm{eq}}$ is equal to the longitudinal normal strain $\varepsilon_{11}$,

(1) $$\varepsilon_{1}^{\mathrm{eq}}=\left|\varepsilon_{11}\right|,$$

while the transverse (matrix) equivalent strain $\varepsilon_{2}^{\mathrm{eq}}$ accounts for the interaction of transverse tensile and shear strains $\varepsilon_{22}$ and $\gamma_{12}$, respectively, such that

(2) $$\varepsilon_{2}^{\mathrm{eq}}=\mathrm{sign}\left(\varepsilon_{22}\right)\sqrt{{\left(\varepsilon_{22}\right)}^{2}+{\left(\frac{\gamma_{12}}{2}\right)}^{2}}.$$

Note that the sign in Equation (2) indicates load cases with a compressive (negative) or tensile (positive) nature.

In local CDM approaches, damage is evaluated on the basis of local strains. In contrast, nonlocal methods consider the state of strain in a finite neighbourhood [30]. The CODAM2 is equipped with a nonlocal averaging scheme so that the local equivalent strains $\varepsilon_{1}^{\mathrm{eq}}$ and $\varepsilon_{2}^{\mathrm{eq}}$ are averaged over a spherical zone $\Omega_{R}$ with radius *R* such that

(3) $${\bar{\varepsilon }}_{\alpha }^{\mathrm{eq}}=\frac{1}{W}\int_{\Omega_{R}}\varepsilon_{\alpha }^{\mathrm{eq}}\left(X\right)w\left(∥X-x∥\right)\;d\Omega_{R},\;\alpha =1,2,$$

where *w* and $W=\int_{\Omega_{R}}w\;d\Omega_{R}$ are the pointwise weight functions and their summation over all points within $\Omega_{R}$, respectively. The norm $∥X-x∥$ measures the distance of the local point ***X*** to every point ***x*** in its neighbourhood within $\Omega_{R}$ [31]. The nonlocal averaging radius *R* introduces a length scale that can be associated with the simulated damage height [32]. In the following, the averaging scheme in Equation (3) is either de-activated or activated for the application of local and nonlocal computations, respectively.

Considering critical strains for damage initiation $\varepsilon_{\alpha }^{i}$ and damage saturation $\varepsilon_{\alpha }^{s}$, CODAM2 damage variables $\omega_{\alpha }$ are determined by

(4) $$\omega_{\alpha }=\left(\frac{\left|\varepsilon_{\alpha }^{\mathrm{eq}}\right|-\varepsilon_{\alpha }^{i}}{\varepsilon_{\alpha }^{s}-\varepsilon_{\alpha }^{i}}\right)\left(\frac{\varepsilon_{\alpha }^{s}}{\varepsilon_{\alpha }^{\mathrm{eq}}}\right)\;\mathrm{for}\;\left(\left|\varepsilon_{\alpha }^{\mathrm{eq}}\right|-\varepsilon_{\alpha }^{i}\right)>0.$$

Note that Equation (4) refers to local damage simulation. If nonlocal simulations are considered, ${\bar{\varepsilon }}_{\alpha }^{\mathrm{eq}}$, ${\bar{\varepsilon }}_{\alpha }^{i}$ and ${\bar{\varepsilon }}_{\alpha }^{s}$ calculate the nonlocal damage variables ${\bar{\omega }}_{\alpha }$.

Damage initiation and saturation strains $\varepsilon_{\alpha }^{i}$ and $\varepsilon_{\alpha }^{s}$ (or ${\bar{\varepsilon }}_{\alpha }^{i}$ and ${\bar{\varepsilon }}_{\alpha }^{s}$) are the key parameters for simulating progressive fracture in FRP composites. For a linear softening shape in local CDM, as shown in Table 1, the fracture energy of each ply of the composite material $G_{\alpha }^{f}$ is related to the damage saturation strain $\varepsilon_{\alpha }^{s}$ and ply strength ***X*** by means of

(5) $$\varepsilon_{\alpha }^{s}=\frac{2G_{\alpha }^{f}}{Xl^{*}}.$$

Ply-based fracture energy values $G_{\alpha }^{f}$ (α=1,2) are challenging to determine. Although compact tension/compression tests can be conducted [33,34] to measure the fracture energy of the laminate, it is not clear how this measurement contributes to the corresponding ply-based values, in particular, to the energy $G_{1}^{f}$ in the fibre direction. Sophisticated in situ measurements and analysis techniques such as CT scans [35] or digital image correlation [36] can be employed to obtain further insight into the damage evolution of individual plies based on observed macroscopic damage at the laminate level. The physical interpretation of nonlocal damage parameters ${\bar{\varepsilon }}_{\alpha }^{i}$ and ${\bar{\varepsilon }}_{\alpha }^{s}$ is even less intuitive. This motivates the use of a data-driven calibration process to determine those parameters.

The damage variables are incorporated into the constitutive behaviour by means of stiffness reduction factors $R_{1}$ and $R_{2}$ such as

(6) $$R_{\alpha }=\left(1-\omega_{\alpha }\right),\;\alpha =1,2.$$

The in-plane secant stiffness matrix $Q_{k}$ describing the $k^{\mathrm{th}}$ layer of a laminate is then

(7) $$Q_{k}=\frac{1}{D}{\left[\begin{array}{ccc} R_{1}E_{1} & R_{1}R_{2}\nu_{12}E_{2} & 0\\ \; & R_{2}E_{2} & 0\\ \mathrm{sym} & \; & DR_{2}G_{12} \end{array}\right]}_{k}$$

where $D=1-\left(R_{1}R_{2}\nu_{12}\nu_{21}\right)$ with the moduli $E_{1}$, $E_{2}$ and $G_{12}$ and the major Poisson’s ratio $\nu_{12}$.

Finally, the overall in-plane secant stiffness A describing a laminate consisting of *n* layers is given by

(8) $$A=\sum_{k=1}^{n}T_{k}^{T}Q_{k}T_{k}t_{k}$$

with the thickness $t_{k}$, the $k^{\mathrm{th}}$ layer and the transformation matrix $T_{k}$.

The CODAM2 has the advantage of using only one through-thickness integration point to describe the laminate behaviour (see Equation (8)), making it more efficient compared with other CDM material models that apply ply properties to multiple integration points. Therefore, The CODAM2 is a promising candidate for the coupling with data-driven optimisation procedures such as GA, as outlined in Section 2. Table 1 illustrates a strain-softening curve in compression which is applied in the fibre (α=1) and in the matrix (α=2) direction in each ply of a composite laminate. The elastic input parameters are independent of the underlying CDM method (local or nonlocal) and hence we consider them to be known [28]. The following sections will determine the damage parameters associated with strain softening in local and nonlocal form using the CODAM2. The parameters to be identified are the damage initiation strains $\varepsilon_{\alpha }^{i}$ and damage saturation strains $\varepsilon_{\alpha }^{s}$ in the fibre α=1 and matrix α=2 directions.

### 2.2. Finite Element Model

**Figure 1:**
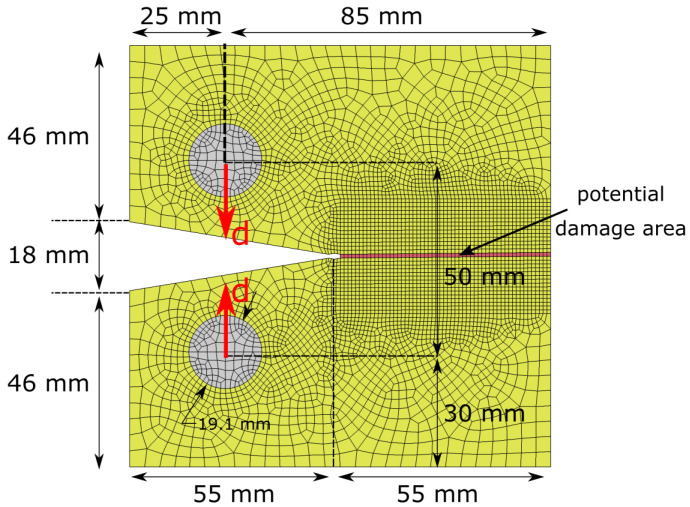
Finite element model to simulate IM7/8552 CFRP laminates subjected to compact compression test for the GA-based calibration of damage input parameters.

To calibrate the damage parameters in compression, we will simulate the compact compression (CC) test shown in Figure 1 [33]. The material of interest is IM7/8552 carbon-fibre-reinforced polymers (CFRP) in quasi-isotropic ${\left[90/45/0/-45\right]}_{4s}$ laminates. The FE model consists of shell elements with an applied laminate thickness of 4 mm. In the vicinity of expected damage growth around the initial notch of the CC specimen, a 1 mm × 1 mm mesh is applied. In the nonlocal simulation, we will apply an averaging radius R=1.5 mm. The advantage of the CODAM2 is that only one shell element through the thickness is required to simulate the quasi-isotropic CFRP laminate by assigning different ply orientations within the material card. A prescribed displacement is applied to the loading pins in the opposite direction to initiate compressive progressive crushing in the highlighted potential damage area shown in Figure 1.

### 2.3. Genetic Algorithms

The previously described FE model of the CC test will be coupled to GA in the commercial optimisation software LS-OPT (as part of Ansys 2021 R2). Given the vector of unknown input parameters $x=\left(\varepsilon_{1}^{i},\varepsilon_{1}^{s},\varepsilon_{2}^{i},\varepsilon_{2}^{s}\right)$, we aim to minimise the Mean Squared Error (MSE) between experimentally measured and FE-based simulation results by

(9) $$\min f_{\mathrm{MSE}}\left(x\right)\;\;\mathrm{subject}\;\mathrm{to}\;\;g_{m}\left(x\right)\geq 0,$$

where $f_{\mathrm{MSE}}\left(x\right)$ is the difference between experimental and simulation results obtained from FE input parameters x. Furthermore, $g_{m}\left(x\right)$ represents constraint functions. Equation (9) will be solved by GA with appropriate settings for selection, crossover and mutation.

**Figure 2:**
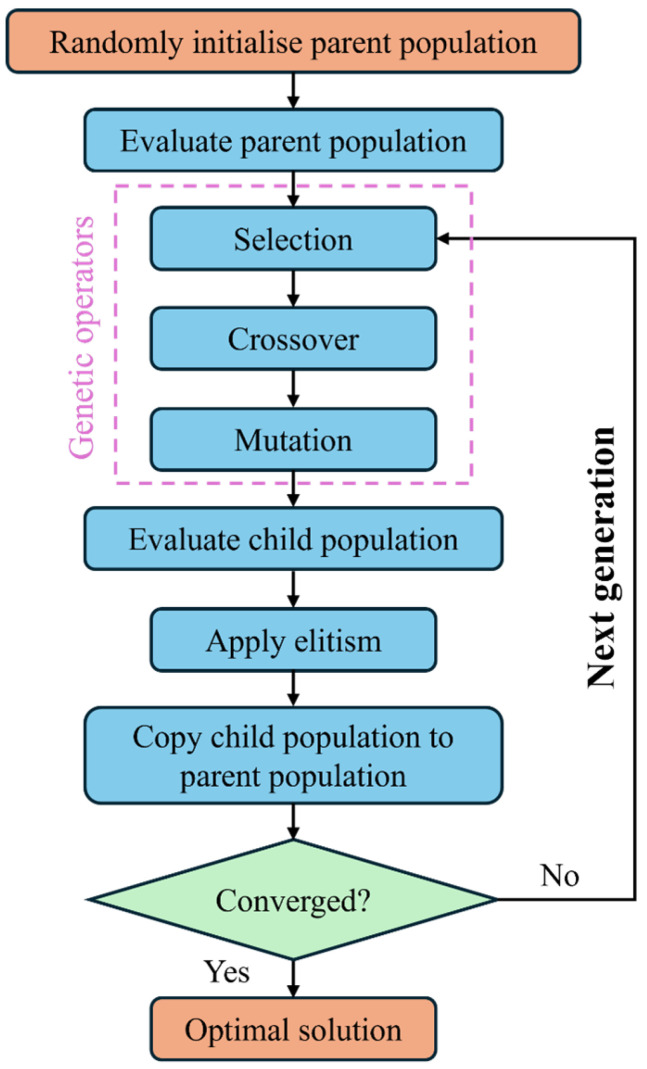
Flowchart of the optimisation process to solve Equation (9) using genetic algorithms.

As illustrated by the flowchart shown in Figure 2, the GA optimisation process begins by generating an initial population of 24 random individuals, each representing a potential solution for calibrating damage parameters in the CFRP laminates. Each individual is evaluated through FE simulations (i.e., 24 FE simulations are evaluated in one generation). The GA select individuals for reproduction based on their fitness. The crossover operation chooses two parents based on their fitness and then exchanges parameters between two parents to create offspring. Afterwards, the mutation operation introduces small random changes to the offspring’s parameters, which helps maintain genetic diversity and prevents the GA from converging prematurely to suboptimal solutions. It is noted that elitism is used in the GA to ensure that the best individuals from the current generation are preserved and passed on to the next generation. This iterative process continues through successive generations until the MSE stabilises, at which point the optimised damage parameters that best match the experimental data are identified. More specific details about these settings can be found in the related literature [28]. This optimisation of the GA will be applied separately to find local and nonlocal input parameters, respectively.

Explicit constraints $g_{m}\left(x\right)$ help to enforce physically meaningful optimisation results. Here, we apply the inequality constraint $g_{1}\left(x\right)\geq 0$ with $g_{1}\left(x\right)=1600-E_{1}\varepsilon_{1}^{i}$ so that the optimised compressive strength in the fibre direction is less than or equal to 1600 MPa based on experimental measurements of 1590 MPa [37].

## 3. Validation: Open-Hole Compression Tests

**Figure 3:**
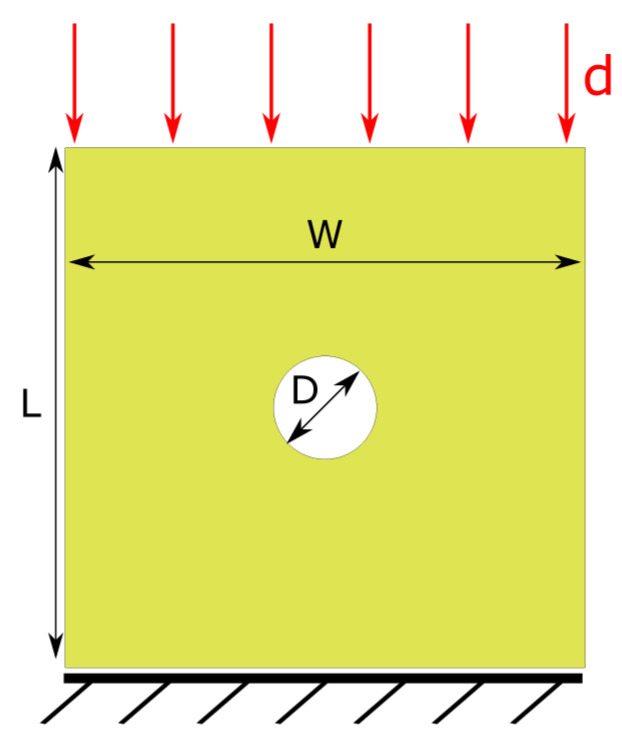
Finite element model to simulate open-hole compression tests for validation of optimised input parameters.

Although the optimisation constraint applied in the previous section on GA ensures that physically meaningful input parameters are obtained from the automated and objective GA calibration procedure, it is important to validate these results against other experimental tests. Here, we consider open-hole compression (OHC) tests [38] of quasi-isotropic ${\left[45/90/-45/0\right]}_{4s}$ IM7/8552 CFRP laminates, the same material used for the GA-based calibration with a slightly different stacking sequence. The thickness of 4 mm is identical to the test samples previously described for the CC tests. Figure 3 shows the geometry of an L = 64 mm × W = 64 mm OHC test sample with a centrally located hole of diameter D = 12.7 mm. This will be referred to as the baseline geometry. A prescribed displacement is applied to the top edge of the OHC sample while the bottom edge is fully constrained. It is known that the investigated CFRP laminates behave in a linear elastic manner with brittle failure in these compression tests [38]. Hence, the quantitative analysis of the simulation results will focus on the open-hole strength, which is calculated by dividing the maximum force in the OHC tests by the net cross-sectional area (here, 64 mm × 4 mm).

As part of the validation study, we will investigate the sensitivity of the open-hole strength and damage patterns with respect to the underlying FE mesh using the local and nonlocal form of the CODAM2 with optimised damage input parameters. Thereby, the mesh size and mesh orientation will be altered. Furthermore, the ability to simulate large-scale structures will be investigated in scaled OHC test samples in comparison to the previously described baseline samples.

## 4. Results and Discussion

After presenting the optimised input parameters for the local and nonlocal simulation of progressive damage in CFRP laminates, this section compares the two continuum damage modelling techniques qualitatively and quantitatively in a range of simulated OHC tests. All FE simulations are completed in LS-DYNA on an Intel Xeon Gold 6226R 2.90 GHz processor with 192 GB installed memory.

### 4.1. Identified Input Parameters

Table 1 summarises the optimal damage input parameters based on the local and nonlocal form of the CODAM2 found by solving Equation (9) by means of the previously described GA-based algorithm. In the local case, the optimisation of the GA converges after 13 generations (totalling 13×24=312 FE simulations) with the objective function value of 0.157. Similarly, the nonlocal procedure converges after 12 generations (12×24=288 FE simulations) and an objective function value of 0.205.

**Figure 4:**
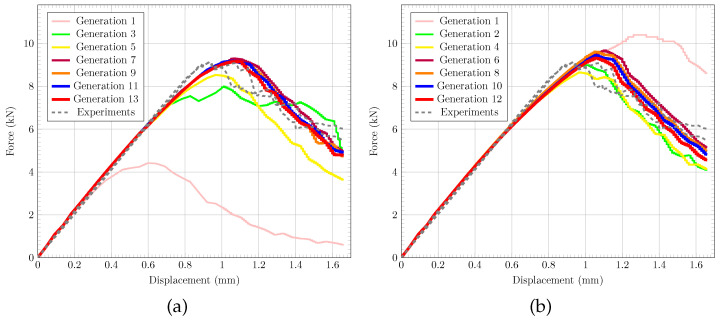
Force vs. displacement results from (**a**) local and (**b**) nonlocal continuum damage models to calibrate damage input parameters with GA. Best FE simulation in generation of GA is shown to visualise convergence towards experimentally measured data [33].

**Figure 5:**
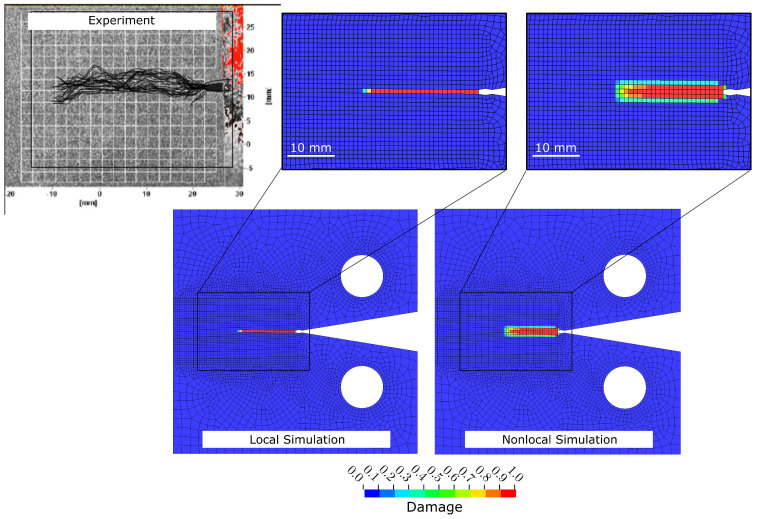
Comparing damage patterns between experiments [33] and local and nonlocal simulation results in the 0° ply with the optimal input parameters at the end of the compact compression tests of ${\left[90/45/0/-45\right]}_{4s}$ IM7/8552 CFRP.

Figure 4 shows the force vs. displacement graphs obtained from CC tests [33] compared to the best FE results of each generation from the local (Figure 4a) and nonlocal (Figure 4b) simulations, respectively. It can be seen that, in both cases, the force vs. displacement graphs converge towards the experimentally measured data, capturing the peak and post-peak behaviour. Note that the best FE results from the first generations in the local and nonlocal models differ significantly, while later generations converge consistently towards the experimental data. This initial difference is due to the random selection of individuals in Generation 1, as outlined in Section 2.3. Figure 5 further demonstrates that both the local and nonlocal FE simulations of the CC tests generate damage patterns that qualitatively agree with observations from experiments [33]. It can be seen that damage in local simulations is confined to a (local) narrow band while the nonlocal simulations produce more realistic damage bands. Overall, the results in Figure 5 show that the GA-based optimisation solely based on force vs. displacement data can calibrate CDM material models, leading to physically meaningful damage progression in FRP composites.

### 4.2. Mesh Size Sensitivity

The optimised input parameters shown in Table 1 are applied to OHC tests using three different mesh sizes (medium/fine/very fine) to investigate the sensitivity of local and nonlocal simulations with respect to the element size. The medium FE mesh with an in-plane element size of 1 mm × 1 mm is consistent with the mesh that we used for the simulation of CC tests in the GA-based calibration procedure. This results in 4855 shell elements and 4931 nodes. The fine mesh consists of a 0.5 mm × 0.5 mm mesh in the expected damage zone, totalling 19,388 shell elements and 19,367 nodes. Lastly, the very fine mesh uses elements of 0.25 mm × 0.25 mm, resulting in a total of 77,878 shell elements and 77,291 nodes.

Mesh size sensitivity is one of the major drawbacks of local CDM methods. Bazant’s crack band scaling [39] offers a simple and efficient solution where the fracture energy $G^{f}$ is scaled according to the characteristic element length $l^{*}$ such that

(10) $$g^{f}=\frac{G^{f}}{l^{*}},$$

where $g^{f}$ is the fracture energy density (area under the stress–strain curve shown in Table 1). Note that $l^{*}=1$ mm in the medium-size mesh, and hence $g^{f}=G^{f}$.

**Figure 6:**
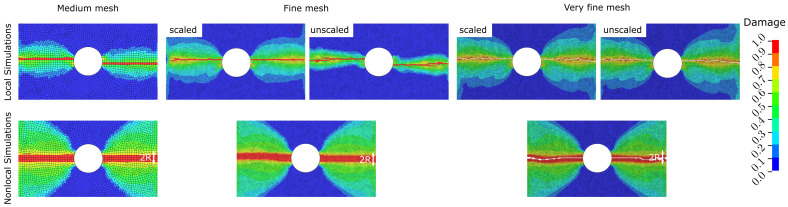
Qualitative comparison of damage in ${\left[45/90/-45/0\right]}_{4s}$ IM7/8552 CFRP laminates after local and nonlocal FE simulation of open-hole compression tests in medium, fine and very fine meshes. The shown damage variable ranges from 0.0 (no damage) to 1.0 (saturated damage) and refers to the 0° ply with fibres oriented in the vertical direction.

Table 2 shows the simulated open-hole compressive strength values for the three FE meshes compared to experimental measurements [38]. The fine and very fine meshes are evaluated with and without the application of crack band scaling, outlined in Equation (10), here referred to as scaled and unscaled simulations, respectively. The results in Table 2 show that the nonlocal simulations yield consistent OHC strength values that are 6–8% below the experimental measurements, irrespective of the mesh size. In contrast, the OHC strength values predicted by local simulations vary significantly. For the scaled version, simulation errors range from 9% to 13% above experimental measurements, while unscaled simulation results are 7–11% below experimentally determined strength values. The error increases with decreasing element size in these cases. More details about this finding will be discussed in Section 4.5. Figure 6 shows the simulated damage in the 0° ply of the ${\left[45/90/-45/0\right]}_{4s}$ laminate around the open hole using the three different mesh sizes. The damage variable equal to one indicates fully saturated damage. As the mesh is aligned in the expected direction of damage growth normal to the applied compressive load, all simulations yield consistent damage bands. One difference is the simulated damage height, as observed in the simulation results of the CC tests shown in Figure 5. Damage localises into one row of elements in the local simulation (hence the need for Bazant’s crack band scaling), whereas the damage height in nonlocal results is constant and equal to the nonlocal averaging diameter (2×R).

The nonlocal simulations show mesh-size-independent results. It should be noted that the activation of nonlocal averaging increases the computational cost as neighbouring integration points are taken into account [40]. The nonlocal simulation of OHC tests took approximately three times longer compared to local counterparts. For example, the simulation with the medium FE mesh completed in 9 min and 27 min using the local and nonlocal features, respectively.

### 4.3. Mesh Orientation Sensitivity

While Bazant’s crack band scaling, shown in Equation (10), is effective in FE meshes that are aligned with the expected direction of damage growth, it cannot overcome the limitation of CDM models in aligning the damage growth direction with the FE mesh in general.

**Figure 7:**
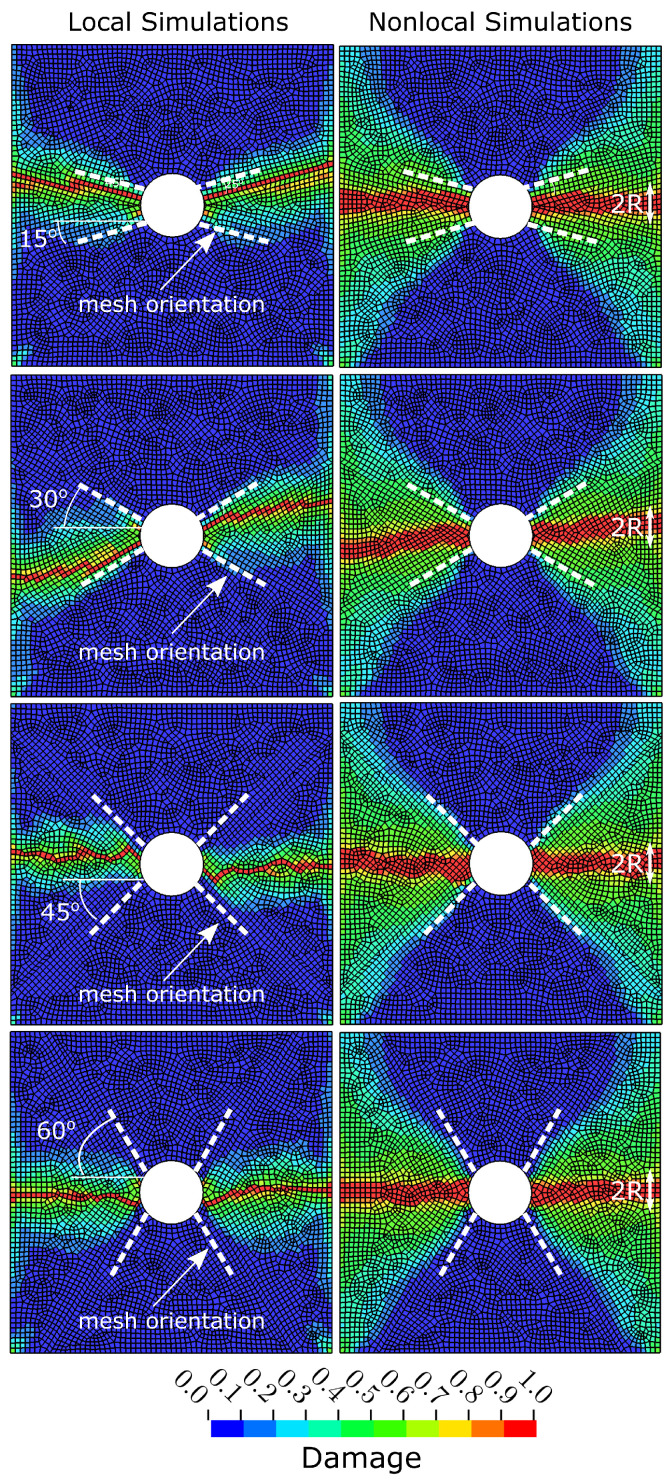
Comparison of simulated damage in 0° ply after open-hole compression tests of ${\left[45/90/-45/0\right]}_{4s}$ IM7/8552 CFRP laminates from the local and nonlocal methods using differently inclined mesh orientation around central hole.

To investigate the sensitivity with respect to mesh orientation, we simulated the OHC test with inclined medium meshes (1 mm × 1 mm) around the open hole. Figure 7 shows the simulated damage from local and nonlocal FE simulations using inclined meshes from 15° up to 60° in increments of 15°. These results show that damage growth in local simulations can be affected by the orientation of the FE meshes. Damage growth aligns with the 15° and 30° inclined FE mesh, resulting in unrealistically inclined damage bands. In contrast, damage bands obtained from nonlocal simulations are not sensitive to the underlying FE mesh, with realistic damage growth being normal to the applied load, as observed in experimental investigations [38]. Again, the damage height in nonlocal simulations is proportional to the applied averaging radius (2×R).

The quantitative analysis of the simulated OHC strength in these inclined meshes shown in Table 3 confirms the findings from Section 4.2, where nonlocal simulations yielded mesh-independent strength results that were about 6–8% below experimental measurements. The local counterparts show inconsistent results with OHC strength values, being 0–8% above the experimentally obtained data. The findings highlight the benefits of nonlocal FE simulations in producing mesh-independent predictions of damage growth and strength. In contrast, local CDM methods result in localised damage bands that conform to the underlying mesh structure, which may lead to erroneous strength predictions.

### 4.4. Size Effects

**Figure 8:**
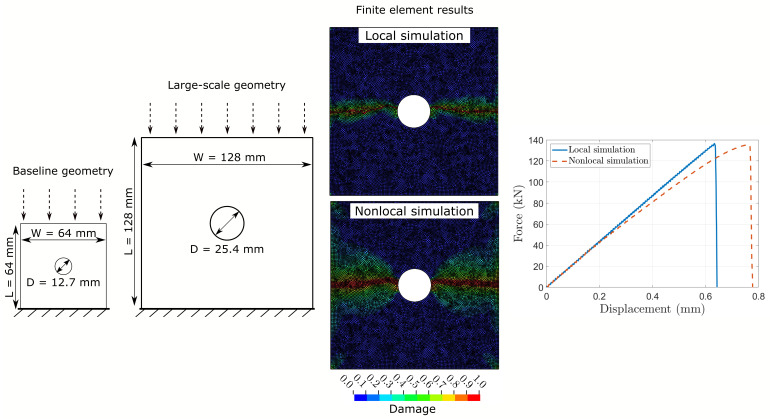
FE results of large-scale open-hole compression test specimen on ${\left[45/90/-45/0\right]}_{4s}$ IM7/8552 CFRP laminates showing qualitative and quantitative comparisons between local and nonlocal simulations. Shown damage refers to the 0° ply of the laminate.

Thanks to its efficiency, CDM promises the simulation of large-scale geometries beyond typical coupon-level specimens without the need to modify any of the FE input parameters. Here, we consider large-scale OHC samples where the in-plane dimensions are doubled compared to the baseline geometry shown in Figure 3. The dimensions are W=L=128 mm with a hole diameter of D = 25.4 mm. Figure 8 illustrates these dimensions and compares the quantitative and qualitative results from local and nonlocal simulations. The FE element size is 1 mm × 1 mm. The local simulation completes in 55 min. Similar to previous results discussed for the baseline OHC geometry in Section 4.2, the nonlocal simulation takes approximately three times longer.

Both simulations, local and nonlocal, yield OHC strength values below the experimental measurements $X_{\mathrm{EXP}}=285$ MPa [38]. The OHC strength from the local $X_{\mathrm{local}}=267$ MPa and nonlocal simulation $X_{\mathrm{local}}=264$ MPa are 6.3% and 7.3% below these experimental results, respectively. These findings are in line with the results discussed in the previous sections on mesh size and mesh orientation dependencies. The nonlocal simulations yield OHC strength values that are consistently 6–8% lower than experimental measurements. In contrast, OHC strength values obtained from local simulations do not show a clear trend. While the local simulations overpredicted the OHC strength by 0–13% in previous analyses, the simulation of the large-scale geometry yields results that underpredict experimental data.

### 4.5. Challenges and Limitations

Both local and nonlocal CDM methods are capable of quantitatively simulating the mechanical response of the quasi-isotropic CFRP composites, as shown by the comparisons to the load vs. displacement curves from the CC tests in Figure 5 and the strength values from the OHC tests shown in Table 2. Intuitively, one would expect the simulation error to decrease with the use of finer mesh sizes. However, Table 2 reveals the opposite trend, with errors increasing as mesh sizes become finer. This finding can be explained by the highly complex evolution of damage in CFRP composites under compressive loading, where different failure modes occur at various instants and length scales. More sophisticated computational models are necessary to represent these failure modes more accurately. For example, stacked elements through the thickness of the laminate could be connected by cohesive elements/surfaces to enable the simulation of delamination via the CZM. Note that more advanced modelling techniques increase the number of required input parameters and increase computational costs, preventing the coupling with data-driven schemes such as GA.

The presented methodologies provide a general framework for calibrating FE input parameters, if the FE simulations can represent the predominant failure modes. A change in the layup sequence of the laminate or in the CFRP material itself would require a re-calibration. Layup or material changes may influence the presence of different failure modes. In instances where alternative failure modes, such as delamination, predominate, it is important to enhance the FE model by incorporating more sophisticated features, such as the CZM.

With the rise of machine learning (ML), FE parameter calibration can be conducted using deep learning methods (e.g., [16,27]). However, ML relies on large datasets. Previous studies have demonstrated that 3000 to 5000 simulations are required to find FE input parameters via ML. This requirement significantly exceeds the number of FE simulations needed within the presented GA framework. The calibration results in Section 4.1 show that approximately 300 FE simulations are sufficient for GA to identify suitable FE input parameters. This finding is promising, as it offers the potential to explore more advanced and computationally demanding damage modelling and simulation methods within a data-driven calibration process using GA.

## 5. Conclusions

This paper presents a comparison between local and nonlocal continuum damage mechanistic finite element models to simulate progressive damage evolution in carbon-fibre-reinforced polymers subjected to compressive loadings. Genetic algorithms enable the objective and automated determination of damage input parameters for the two analysis types. It is found that the nonlocal simulations yield consistent damage evolution and open-hole strength values that are 6–8% below experimental measurements. In contrast, the results from local simulations confirm the typical limitations of this method with mesh-dependent damage growth and simulated strength values that are sensitive to the underlying mesh size and mesh orientation. The presented combination with genetic algorithms enhances the transparency and facilitates the use of nonlocal methods to simulate progressive damage in composite materials.

## Figures and Tables

**Table 1:** Summary of FE input parameters in fibre (α=1) and matrix (α=2) directions to simulate progressive damage evolution of IM7/8552 CFRP composites subjected to compressive loading.

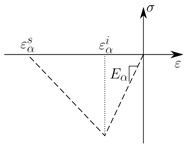		**Elastic input parameters** [28]			
		$E_{1}$ (GPa)	$E_{2}$ (GPa)	$G_{12}$ (GPa)	$\nu_{12}$ (-)
		154.6	8.96	4.69	0.31
		**Strength/damage input parameters**			
		$\varepsilon_{1}^{i}$ (-)	$\varepsilon_{1}^{s}$ (-)	$\varepsilon_{2}^{i}$ (-)	$\varepsilon_{2}^{s}$ (-)
	Local	0.00676	0.143	0.017	0.0194
	Nonlocal	0.006	0.0872	0.00114	0.0171

**Table 2:** Simulation results for open-hole compressive strength in MPa of IM7/8552 CFRP laminates using different mesh sizes (medium/fine/very fine). Difference from average experimental results of 300 MPa [38] is shown in square brackets.

		Medium	Fine	Very Fine
Local simulations	Scaled	307 [+2.3%]	327 [+9.0%]	339 [+13.0%]
	Unscaled		278 [−7.3%]	267 [−11.0%]
Nonlocal simulations		281 [−6.3%]	278 [−7.3%]	276 [−8.0%]

**Table 3:** Simulated open-hole compressive strength results in MPa of IM7/8552 CFRP with inclined mesh orientations around central hole. Difference to average experimental results of 300 MPa [38] is shown in square brackets.

	**15°**	**30°**	**45°**	**60°**
Local simulations	323 [+7.7%]	319 [+6.3%]	300 [± 0.0%]	325 [+8.3%]
Nonlocal simulations	279 [−7.0%]	280 [−6.7%]	280 [−6.7%]	282 [−6.0%]

## Data Availability

Data will be made available upon request.
